# Psychiatric symptoms as a clinical presentation of Cushing’s syndrome

**DOI:** 10.1186/1744-859X-12-23

**Published:** 2013-07-17

**Authors:** Alice Tang, Anthony J O’Sullivan, Terry Diamond, Andrew Gerard, Peter Campbell

**Affiliations:** 1 Department of Endocrinology, St George Hospital, Gray St, Kogarah, New South Wales, Australia; 2 Department of Medicine, St George Hospital and UNSW Medicine, University of New South Wales, Kensington, New South Wales, Australia; 3 Department of Consultation-Liaison Psychiatry, St George Hospital, Kogarah, New South Wales, Australia; 4 Department of Surgery, St George Hospital, Kogarah, New South Wales, Australia

**Keywords:** Hypercortisolaemia, Hypercortisolism, Cushing’s syndrome, Psychosis, Depression, Catatonia

## Abstract

Cushing’s syndrome can present with a spectrum of symptoms; however, it is less recognised that psychiatric symptoms can form part of the clinical presenting features. In the investigations for an organic cause for a psychiatric illness, Cushing’s syndrome needs to be considered, especially if there are other features such as hirsutism or hypertension. In this article, the two cases reported demonstrate that a prompt diagnosis is not only important for psychiatric management but also crucial for timely institution of the necessary treatment of life-threatening causes of hypercortisolaemia such as metastatic adrenal carcinoma.

## Background

Endogenous Cushing’s syndrome results from chronic excess glucocorticoid production from the adrenal glands. It may be adrenocorticotropic hormone (ACTH) dependent (80%–85%) or independent (15%–20%). ACTH dependent causes include, most commonly, a pituitary corticotrophic adenoma (Cushing’s disease), less frequently, an extra-pituitary tumour (ectopic ACTH syndrome) and rarely a tumour secreting corticotropin-releasing hormone (CRH). ACTH independent causes are from unilateral adrenocortical tumours, which can be benign or malignant, or bilateral adrenal hyperplasia [1].

Hyper-vigilance is necessary because patients can present with features that can be nonspecific and insidious in development, and the disease activity can vary over time. The non-psychiatric clinical features of Cushing’s syndrome can include central adiposity, proximal myopathy, thin skin, purple striae on the trunk, scalp hair loss, fatigue, hypertension, glucose intolerance, acne, hirsutism and menstrual irregularity. The clinical features of Cushing’s syndrome can also be typical of other common disorders such as the metabolic syndrome, with insulin resistance, and polycystic ovary syndrome.

Depression, mood dysregulation, sleep disturbance and cognitive abnormalities are also observed in Cushing’s syndrome [1]. The rates for each of these symptoms vary widely across studies. Depression is the most prevalent psychiatric disturbance in Cushing’s syndrome. A major depressive syndrome is seen in 50%–70% of the cases [2]. Other associated features include anxiety in 12%–79% of the cases [3], as well as a rate of 3% for hypomania [4]. Less common are features of psychosis and mania [5,6].

Reassessment of some of these patients after the reduction of cortisol levels showed a reduced rate of psychiatric diagnoses, with improvements in depression, anxiety, somatic symptoms and neuroticism scores [4].

Another prospective study of 33 patients with active Cushing’s syndrome showed that 67% had psychopathology before cure. Of these, 52% had atypical depressive disorder and 12% had major depressive disorder. One year post-cure, overall psychopathology improved to 24% of the subjects with atypical depressive disorder remaining the most common diagnosis [7]. The authors report that a premorbid or concurrent psychiatric diagnosis with Cushing’s syndrome did not influence the incidence of psychopathology after the correction for high cortisol levels [7]. Some patients have been reported to have residual psychiatric symptoms and cognitive deficits long after the cure of Cushing’s syndrome [1].

Conceptual models have been reported to try to explain glucocorticoid actions in the brain. Psychiatric features of Cushing’s syndrome are associated with increased net glucocorticoid signalling via the glucocorticoid receptors. It has also been suggested that the memory deficits are related to dysfunction of the neocortex and hippocampus, areas with high density of glucocorticoid receptors. Interestingly, the hypotheses relating to hypercortisolaemia associated with depression are less straight forward. Here, the glucocorticoid signalling *insufficiency* hypothesis suggests that hypercortisolaemia is an attempt to overcome primary glucocorticoid receptor resistance related to down-regulated glucocorticoid receptors. In contrast, the glucocorticoid signalling *overactivity* hypothesis for depression suggests there is overall increased net signalling via the glucocorticoid receptor. Changes in cortisol activity leading to decreased serotonin and increased dopamine cerebral activity have also been described [8].

Evaluation of patients with suspected Cushing’s syndrome can be complex and involvement of an endocrinologist is recommended. Initial screening tests include more than one elevated 24-h urinary free cortisol level and/or lack of cortisol suppression after low-dose dexamethasone testing. Once hypercortisolaemia is confirmed, ACTH levels, CRH stimulation test, high-dose dexamethasone test and imaging may be considered to differentiate whether the source may be adrenal, pituitary or ectopic [1]. However, it needs to be considered that a primary psychiatric condition may be associated with increased cortisol levels, so false positive findings need to be excluded.

In the present article, we report two cases of psychosis, which were the presenting features of Cushing’s syndrome, suggesting that clinicians should be mindful that Cushing’s syndrome could be a cause of psychiatric symptoms. Psychiatric symptoms may be the original presentation and the diagnosis can easily be missed without a high index of suspicion. Psychiatric symptoms can potentially be refractory to psychotropic medications with untreated hypercortisolaemia, and the underlying cause can have a high mortality and morbidity if left unrecognised.

## Case presentations

### Case report 1

A 46-year-old Asian woman presented to the hospital with major depression with psychotic features, manifesting as auditory hallucinations, depressed mood, anxiety and somatic complaints. This presentation was on a background of polypharmacy overdose 2 months earlier without suicidal intent. There was a gradual decline in the mental state since that discharge. She was non-adherent to olanzapine and citalopram, and previous investigations during recent multiple hospital admissions had not found an organic cause for her psychiatric presentation.

Upon investigations for complaints of headache and chest pains, she was found to have had developed hypertension and hypokalaemia (potassium 2.4 mmol/L). She was also noted to have a cushingoid appearance. Cushing’s syndrome was confirmed with an elevated serum cortisol 858 nmol/L (normal range 155–599 nmol/L) and urinary free cortisol >999 nmol/24 h (normal range 50–170 nmol/day). These findings were confirmed on repeat testing with serum cortisol of 836 nmol/L and urine free cortisol 441 nmol/24 h despite incompletion of the urine specimen. Serum ACTH was suppressed on both occasions (<5 pg/mL) indicating a primary adrenal cause.

Abdominal computed tomography (CT) scan with contrast showed an irregular 3-cm left adrenal lesion with reported low density and no enhancement with contrast. Markedly increased glucose metabolism in the left adrenal gland was found, consistent with a high grade tumour, on fluorodeoxyglucose positron emission tomography/computed tomography (FDG PET/CT) whole body scanning. No other sites of disease were found. Her plasma aldosterone to renin ratio and catecholamines were normal excluding primary hyperaldosteronism and phaeochromocytoma.

She underwent a laparoscopic left adrenalectomy with perioperative hydrocortisone cover. The histological appearance was consistent with an adrenal cortical adenoma. A synacthen test 4 days post-operatively showed persistent adrenal insufficiency due to suppression of the right adrenal gland activity, therefore requiring ongoing prednisone replacement therapy which was withdrawn over the next few months.

By discharge, she had been stabilised with aripiprazole 20 mg mane and mirtazapine 30 mg nocte. By 6 weeks post surgery, her psychosis had resolved and her cushingoid features showed improvement. By 6 months post surgery, she had been weaned off her psychotropic medications and antihypertensive medications, and her mental state and blood pressure were normal.

### Case report 2

A 25-year-old Caucasian woman presented to the hospital with 6 days of psychotic symptoms on a background of no previous psychiatric diagnosis. In the immediate pre-hospitalisation period, she presented primarily with a depressive syndrome with psychotic features (auditory hallucinations and nihilistic delusions). Furthermore, in that previous 6 months, she had suffered a myriad of other symptoms including insomnia, weight loss, virilising features (hirsutism, facial acne and scalp hair loss) and other cushingoid features. A provisional diagnosis of psychosis secondary to a general medical condition was made.

Investigations revealed hypercortisolaemia (serum cortisol 1,225 nmol/L), and ACTH-independent Cushing’s syndrome was confirmed with a persistently elevated cortisol (1,040 nmol/L) with ACTH suppression (1 pg/mL). Ultrasound, CT and magnetic resonance imaging (MRI) showed an 11-cm left necrotic adrenal cortical tumour with a presumed 2.2-cm secondary tumour in the liver. Left upper quandrantectomy (splenectomy, nephrectomy, adrenalectomy and distal pancreatectomy) was performed. Adrenal and liver histology confirmed adrenal cortical carcinoma with local infiltration and distant metastasis.

In the first instance, her psychotropics were quetiapine and venlafaxine. Pre-operatively, her hypercortisolaemia was treated with metyrapone (adrenocorticoid synthesis inhibitor) and mitotane (adrenolytic compound). She continued on low-dose prednisone post-operatively because of partial suppression of the contra-lateral adrenal gland from long-standing endogenous hypercortisolaemia and hypothalamic-pituitary axis suppression.

Post-operatively, the patient went into a catatonic state which lasted a total of 2 weeks. It was characterised by, at various times, immobility, mutism, persecutory delusions and other psychotic features, automatic obedience, staring, impulsivity and waxy flexibility. There was gradual response to the cessation of quetiapine and venlafaxine and to the use of lorazepam (oral/intravenous). With the resolution of the catatonia, she was found to have an underlying depressive syndrome with psychotic features. The above catatonia initially persisted despite normalisation of her serum cortisol (135 nmol/L) and plasma ACTH (8 pg/mL). Other medical causes of catatonia were further excluded with appropriate blood and normal cerebrospinal fluid analyses and cerebral MRI.

At the time of discharge, her psychotic symptoms had resolved and her mood had greatly improved. She was discharged with medications risperidone, modified-release venlafaxine and reducing dose of lorazepam. Psychiatric follow-up was arranged. Unfortunately, 2 months after surgery, during evaluation for adjuvant radiotherapy, she was found to have recurrent hepatic metastasis that required chemotherapy.

## Conclusions

In the present article, we present a case of acute-on-chronic psychotic depression associated with Cushing’s syndrome from an adrenal adenoma and another case of psychosis secondary to Cushing’s syndrome from a metastatic adrenal carcinoma. In both cases, the diagnosis of Cushing’s syndrome was only made when mental disturbance had escalated requiring further medical attention. These cases highlight the importance of the consideration of Cushing’s syndrome as a differential diagnosis when ruling out medical causes in patients with either new or persistent mental disturbance. The physical and biochemical features associated with Cushing’s syndrome can be varied and subtle at times. Early recognition and timely institution of appropriate management can minimise significant morbidity and mortality.

## Consent

Written informed consent was obtained from the patients for publication of these case reports and any accompanying images. A copy of the written consent is available for review by the Editor-in-Chief of this journal.

## Abbreviations

ACTH: Adrenocorticotropic hormone; CRH: Corticotropin-releasing hormone; CT: Computed tomography; FDG PET/CT: Fluorodeoxyglucose positron emission tomography/computed tomography; MRI: Magnetic resonance imaging.

## Competing interests

The authors declare that they have no competing interests.

## Authors’ contributions

AT drafted the manuscript. AO conceived of the case reports, participated in its design, coordination and helped to draft the manuscript. TD contributed to the acquisition, analysis and interpretation of the data. AG participated in its design and helped to draft the manuscript. PC contributed to the acquisition, analysis and interpretation of data. All authors read and approved the final manuscript.
